# The attractions of medicine: the generic motivations of medical school applicants in relation to demography, personality and achievement

**DOI:** 10.1186/1472-6920-6-11

**Published:** 2006-02-21

**Authors:** IC McManus, G Livingston, Cornelius Katona

**Affiliations:** 1 Department of Psychology, University College London, Gower Street, London WC1E 6BT, UK; 2 Department of Mental Health Sciences/Camden and Islington Mental Health and Social Care trust, Holborn Union Building, Archway Campus, University College London, London N19 5NL, UK; 3 Kent Institute of Medicine and Health Science, R&D Building, University of Kent, Canterbury, Kent, CT2 7PD, UK

## Abstract

**Background:**

The motivational and other factors used by medical students in making their career choices for specific medical specialities have been looked at in a number of studies in the literature. There are however few studies that assess the generic factors which make medicine itself of interest to medical students and to potential medical students. This study describes a novel questionnaire that assesses the interests and attractions of different aspects of medical practice in a varied range of medical scenarios, and relates them to demographic, academic, personality and learning style measures in a large group of individuals considering applying to medical school.

**Methods:**

A questionnaire study was conducted among those attending *Medlink*, a two-day conference for individuals considering applying to medical school for a career in medicine. The main outcome measure was the Medical Situations Questionnaire, in which individuals ranked the attraction of three different aspects of medical practise in each of nine detailed, realistic medical scenarios in a wide range of medical specialities. As well as requiring clear choices, the questionnaire was also designed so that all of the possible answers were attractive and positive, thereby helping to eliminate social demand characteristics. Factor analysis of the responses found four generic motivational dimensions, which we labelled *Indispensability*, *Helping People*, *Respect *and *Science*. Background factors assessed included sex, ethnicity, class, medical parents, GCSE academic achievement, the 'Big Five' personality factors, empathy, learning styles, and a social desirability scale.

**Results:**

2867 individuals, broadly representative of applicants to medical schools, completed the questionnaire. The four generic motivational factors correlated with a range of background factors. These correlations were explored by multiple regression, and by path analysis, using LISREL to assess direct and indirect effects upon the factors. *Helping People *was particularly related to agreeableness; *Indispensability *to a strategic approach to learning; *Respect *to a surface approach to learning; and *Science *to openness to experience. Sex had many indirect influences upon generic motivations. Ethnic origin also had indirect influences via neuroticism and surface learning, and social class only had indirect influences via lower academic achievement. Coming from a medical family had no influence upon generic motivations.

**Conclusion:**

Generic motivations for medicine as a career can be assessed using the Medical Situations Questionnaire, without undue response bias due to demand characteristics. The validity of the motivational factors is suggested by the meaningful and interpretable correlations with background factors such as demographics, personality, and learning styles. Further development of the questionnaire is needed if it is to be used at an individual level, either for counselling or for student selection.

## Background

19,944 individuals applied to United Kingdom medical schools in 2003. Surprisingly, very little is known about why they applied to study medicine, what their interests in medicine are, and what is it in particular that they like about the idea of being a doctor and practising medicine.

Medical school selection committees almost invariably try to assess what is broadly called 'motivation', a term remarkably lacking in definition in the literature, and with few studies that consider it in detail. A cliché of the medical student selection interview is the question, "So why do you want to be a doctor?", which often receives the equally clichéd reply, "Because I want to help people". Many applicants to medical school *do *want to help people, but, as Freud and Breuer commented [1] (pp. 289, 346, 376), human behaviour is over-determined (*überbestimmt, überdeterminiert*), with any single event being driven by a host of motivations. Helping people may be what medicine is about, but it is also about helping oneself to be successful, in terms of both status and finance, about enjoying oneself, about being wanted, about power and control, about finding oneself, about understanding people and the world in which they are born, live and die, and about intellectual rewards, particularly in being a medical scientist, as well as a host of more primitive, more basic, motivators. If pushed, most individuals will admit that many or even most of these factors have been influential when they have thought about a medical career, and about becoming a doctor. It is therefore the relative importance given to these various motivations by individual prospective doctors, and their relationship to career choices within medicine, that is of most interest.

Studies of the primary motivations of medical school applicants and medical students are infrequent, typically asking students to rate each of a number of possible reasons for being a doctor on a Likert scale [2,3]. A retrospective analysis of doctors' reasons for entering medicine found five main factors: being good at science subjects, wanting a good interesting career, always having wanted to be a doctor, influenced by friends and relations, and wanting to help or work with people [4]. It is possible that most such factors are important for most people, but there are also demand characteristics which make respondents less likely to rate highly such socially desirable items as "thought it would be glamorous/good life-style/status", or "job security" [4], and even less so for "becoming rich", or "having power over people", however true they may be. That is particularly so if gaining a place depends on the answers to such questions. The present study wished to use a novel type of questionnaire to circumvent some of these problems, firstly by forcing choice between different motivations, and secondly by making all answers socially desirable so that each could be answered positively and without the purposes of the questionnaire being too obvious.

Primary motivations become clearer when possible motivations are in conflict. Helping people and doing scientific research are both admirable motivations, but very often both activities cannot be carried out at the same time, requiring a decision as to which is the more important for a particular doctor. When choices have to be made, motivations become clearer.

The medical education literature contains a large number of studies examining which specialities students or doctors choose, such as surgery, paediatrics, psychiatry, general practice or pathology [5-13]. Medicine provides a rich and varied range of career specialties, differing in the types of patient and the types of clinical problem with which they deal. The immediate job characteristics of a neurosurgeon, a public health physician, a histopathologist and a psychiatrist vary almost as much as being a doctor itself differs from being an airline pilot, an accountant or a museum curator. Specialities differ in the venue in which the work occurs (hospital or community), the organ or tissue involved, the involvement of the patient (conscious, anaesthetised, or as a tissue specimen, or an epidemiological sample), and the time-scale of the doctor-patient interaction (perhaps minutes for a histopathologist, hours and days for a neurosurgeon, or months to years for a GP or psychiatrist). Although such job characteristics will undoubtedly be a part of the explanation of *speciality *preferences, it is also the case that a career in any medical speciality, to some degree, allows a host of approaches to work, and hence for different career-related motivations to be rewarded. For instance, the desire to help people will be satisfied in almost any medical speciality, as will a desire to do medical research. Although of interest, studies of the motivations to specialise in a particular area, or discipline, say little about the *generic *motivations for being a doctor, and every speciality requires firstly that one becomes a doctor. In this paper we are primarily interested in *the *career *of *medicine rather than *a *career *within *medicine.

The questionnaire described here approaches the issue of generic motivation by asking participants to choose what is particularly attractive to them in several medical scenarios involving different specialities and settings. By rank ordering the appeal of three different aspects of a scenario, generic motivational preferences are expressed. Furthermore, all the choices are between aspects of the scenario expressed in a positive way, in order to reduce responses driven mainly by social desirability and expectations. This also allows generic motivations to be expressed more clearly and more obviously. Questionnaires, like medical school interview questions, can be criticised for being too abstract, raising the possibility that different respondents conceptualise the same words and phrases in very different ways. This questionnaire was therefore designed around a series of concrete medical situations described in clinically realistic case vignettes. Each has sufficient detail for respondents to place themselves empathetically in the situation. Investigators have used vignettes as data collection tools since the 1950s, particularly to encourage discussion of difficult topics [14]. Almquist *et al *[15] examined the correlation between answers based on vignettes and an actual medical performance, and found similar responses in both, suggesting that answers to vignettes may be similar to what people might do in equivalent real clinical situations.

Our primary intention in this study was to explore the nature of the generic motivations for studying medicine in those considering medical careers, and to examine how those motivations differed between different types of individual in terms of demography and personality. We were not trying to produce a scoreable instrument for selection or other purposes.

We are aware of a number of current tensions in medical student selection and training, and we chose background variables which might help to illuminate them. As we also wanted to be able to use a path analytic approach to assess direct and indirect influences upon generic motivations, we chose some of our variables on the basis that they had been used before, that relationships between them were known and understood, and that replicating such relationships would help to validate the present data set.

The demography of medical students has been of particular recent interest, with concerns about the increasing proportions of female students [16], and about the problems in medical school of male and ethnic minority students [17]. There has also been a growing awareness that medical students typically come from relatively high social classes [18,19], many from medical families [20-23], and concern that such individuals have different motivations and interests in medicine as a career [24].

The role of personality in understanding individual differences has seen a renaissance in the past two decades, in large part due to a growing consensus of the importance of the 'Big Five' personality factors, of extraversion, neuroticism, openness to experience, agreeableness, and conscientiousness as the canonical dimensions of personality [25]. In particular, all the Big Five personality measures, except openness to experience, have been shown to related to doctors' levels of stress and burnout [26,27]. Other aspects of individual differences have also come to the fore. One of particular interest in the context of generic motivations for studying medicine, concerns empathy, which might be expected to underpin aspects of the doctor-patient relationship [28,29]. A high level of academic performance at medical school is necessary to achieve adequate levels of knowledge and competence. Previous academic achievement at examinations and the learning styles and study habits of students predict success [30,31]. In addition, the area of achievement may relate to motivation and attitudes, with students having more experience or higher grades in humanities subjects being more patient-orientated [32-34]. Our background factors therefore included measures of personality, empathy, stress, learning styles, and academic achievement in different subjects, in order to assess the extent and the nature of the influences on generic motivations.

## Method

### Participants

Students attending, *Medlink*, a national sixth form conference for prospective medical students, in December 2003, were asked to take part in the study. Further details of the conference can be found on the internet [35]. Each was given a copy of the questionnaire and a letter explaining the study, as they approached the lecture theatres. Questionnaires were collected after the lectures had finished. A copy of the questionnaire is provided as Section A of Additional File 1. The covering letter emphasised that all information would be confidential and that universities to which applicants were applying would have no knowledge either of their participation or the answers they had given. Students were asked to give an immediate response to each question, rather than spending a long time thinking about each response.

### Measures

The questionnaire included a number of separate measures:

1. **Demographics **(questions 10–15). Candidates provided information about their sex, year of birth, parents' occupational grouping, and whether either parent was a doctor. In addition questions were included about ethnic origin and religion using 2001 UK Census phraseology (see the census form for England [36], and the questions recommended by the UK Commission for Racial Equality [37], which are very similar to those on the census form).

2. **Academic qualifications **(questions 7 and 8): Candidates provided information about school exams taken and about to be taken. They had all completed their GCSE (General Certificate of School Education) examinations, which are usually taken in the academic year the pupil turns 16 and cover a wide range of subjects. Students provided grades in each subject, which range from A* and A through to G, as well as saying whether they had taken or were taking AS (Advanced Subsidiary) level exams or A (Advanced) level examinations, the standard exams for entry to university. Most participants in the present study were pre-A/AS level, so only GCSE results are analysed here. GCSEs were summarised by the number of subjects taken, the total points scored (A* = 6, A = 5, B = 4, etc), the number of A* grades, and the mean grade. Similar scores were calculated separately for science GCSEs and non-science GCSEs, and a score was also calculated which was the ratio of the mean grade at science GCSEs to the non-science GCSEs. GCSE scores were set as missing in those candidates taking fewer than 5 GCSEs, since there were usually special reasons in this minority of candidates (such as being from overseas).

Summary measures were also computed for the number of science AS-levels, the number of non-science AS-levels, the total number of AS-levels being taken, and the proportion of AS-levels which were science subjects. Data were excluded for individuals taking three or fewer AS-levels as these were rare, and generally had special circumstances.

3. **Individual difference measures**. Several standard questionnaires used in previous studies were included to assess aspects of individual differences in personality and study habits.

a. **Study habits and learning styles **(Question 3). These were assessed using the shortened Study Process Questionnaire [38], which provides measures of Surface, Deep and Strategic (Achieving) learning styles and motivations.

b. **Personality **(Question 4). Personality was assessed using an abbreviated Big Five measure, used in a number of previous studies [26,27], which provides measures of Neuroticism, Extraversion, Openness to Experience, Agreeableness and Conscientiousness.

c. **Empathy **(Question 5). Empathy was assessed using an abbreviated version of Davis' Interpersonal Reactivity Index [28,39,40], which provides four sub-scales of Fantasy (the ability to fantasise about the personal impact of different emotional situations), Perspective-taking (the ability to see a situation through someone else's eyes), Empathic concern (the extent to which the emotional problems of others are things which concern and worry one), and Personal distress (the extent to which one suffers oneself when others are themselves distressed).

d. **Social desirability scale **(Question 5). The last three items of question 5 were derived from high loading items of the generic social desirability scale developed by Merrill *et al *[41], which is based on an abbreviated version of the Marlowe-Crowne Social Desirability scale [42]. High scorers are those who say that they always admit it when they make a mistake, are always a good listener, and never feel resentful when they don't get their own way.

e. **Stress **(Question 6). Stress levels were assessed using the 12-item version of the General Health Questionnaire (GHQ-12), which has been used in other studies of stress in doctors [26,27].

f. **Career Preferences **(Question 2). Candidates were asked to indicate their interest in 21 different medical specialities as possible careers.

4. **The Medical Situations Questionnaire (MSQ) **(Question 1). This is a new questionnaire, piloted over several years on small groups of medical school applicants, and then piloted extensively in a previous *Medlink survey *in 2002. A summary of the psychometric properties of the current version can be found in the Results section below, and also Section B of Additional file 1. The questionnaire describes vignettes covering nine medical specialities (surgery, psychiatry, radiology, hospital medicine, general practice, obstetrics and gynaecology, anaesthetics, research and public health). Three aspects of the doctor's role were described after each situation e.g. in situation 3, knowing that the patient's treatment will depend entirely on your diagnostic skills; being in a well-paid and well-respected job with fixed hours; knowing that you have helped someone by providing an effective service. Participants rank ordered each of the three aspects in each vignette, from most appealing to least appealing.

Finally, and after the aspects of each individual vignette had been ranked, respondents rank ordered the vignettes themselves, thereby expressing a preference for, say, surgery, public health medicine, psychiatry, or clinical research, preferences expressed after deep and active processing of specific aspects of each speciality, making them more likely to be valid preferences.

### Statistical analysis

Conventional statistical analysis used SPSS v11.5. Path analysis used LISREL 8.52. Missing values were handled by pairwise deletion for simple statistics, mean substitution for the factor analysis, and the SPSS EM algorithm for data analysed with LISREL.

### Ethics

The proposed study was shown to Camden and Islington Community Health Services NHS Trust and they confirmed that formal ethical permission was not required.

## Results

### Participants and data

The questionnaire was returned by a total of 2867 individuals.

### Sex

1014 participants were male (37.4%), 1697 (59.2%) were female, and sex was not stated for 156 (5.4%) participants.

### Age

The modal age when attending the conference was 16 years (mean = 16.44; median = 16; SD = .77; range 14 – 30), with only 1.0% being aged 18 or over.

### Ethnic origin

The sixteen categories of ethnic origin specified in the questionnaire were reduced to five standard categories. 2090 participants were White (73.9%), with 737 being non-White (26.1%), of whom 455 (16.1%) were Asian or Asian British, 144 were Chinese or Other ethnic group (5.1%), 58 were Black or Black British (2.1%), and 80 were Mixed (2.8%). 40 participants did not give their ethnic origin. In general, statistical analyses will compare White with non-White applicants, with more detailed analyses restricted to cases of particular interest.

### Social class

Applicants described the social class of their parents using a modified form of the pre-2002 social class categories of the Registrar-General. Father's social class is used for most official purposes, and we did that here. 1503 (52.4%) were from social class I (professional), 788 (27.5%) from social class II, 331 (12.3%) from social III, 36 (1.3%) from social class IV, and 26 (0.9%) from social class V (Manual unskilled), with 183 (6.4%) either not providing information or replying unclassifiable. For convenience in interpreting correlations and other statistical analyses, the classes were coded as I = 5, II = 4, III = 3, IV = 2, and V = 1, so that higher social class coincides with higher numerical values.

### Medical families

265 participants reported that only their father was a doctor, 76 that only their mother was a doctor, and 128 that their mother and their father were both doctors. Overall, therefore, 469 participants (16.4%) were from medical families.

### Missing data

Of the 116 variables in questions 1 to 6, 5068 data points were missing. This represented only a small proportion, 1.52%, of the 332,572 possible data points.

### The Medical Situations Questionnaire

The Medical Situations Questionnaire provides the major outcome variables of the study, and it is described in summary here and in detail in Section B of Additional File 1, where detailed descriptive statistics can be found, along with factor loadings, scree plot and other technical details of the factor analysis. The major interest was in the factor structure of the questionnaire, and in particular in extracting independent measures of the different motivations and interests in becoming a doctor.

Factor analysis used a principal components analysis for extraction, after which a Varimax rotation was used to achieve simple structure. Factor scores were extracted using the regression method.

The scree-slope analysis provided evidence for four separable factors, with a distinct break in the scree-slope between four and five factors. Extraction of four factors provided clearly interpretable factors, whereas extraction of five factors found that the fifth factor loaded on only two measures and could not readily be interpretable. The four factors were identified as follows, with most of the reification depending on loadings which were positive rather than negative, since they are easier to interpret.

1. Factor 1: *Indispensability*. This factor was characterised by the importance of make the decision to operate (situation 1), being the leader of the team (situation 2), treating a life-threatening emergency (situation 3), playing the crucial role in diagnosis (situation 4), and being able to do a Caesarean section (situation 9), and by a preference for the emergency surgery of situation 1 and the complex anaesthetic requirements of situation 8. We have labelled this factor *Indispensability*, although it perhaps also has components of control, power and technical expertise.

2. Factor 2: *Helping people*. The five positive loadings on this factor showed the importance of helping to alleviate a patient's social problem (situation 2), helping someone by providing an effective service (situation 3), helping to use public funds for preventing cancer (situation 6), helping people with heart disease by doing research (situation 7), and creating a situation whereby women deliver babies safely and naturally (situation 9). We have labelled this factor *Helping People*, although it could also be called Caring, Compassion, Support or Service.

3. Factor 3: *Respect*. The positive loadings on this factor emphasise the importance of being respected for the ability to counsel patients (situation 2), being in a well-paid, respected job (situation 3), being respected and trust by the patient's family (situation 5), knowing you are respected because of publishing in a top medical journal (situation 7), and having technical skills that will always be in demand (situation 8). This factor has been labelled *Respect*, since that term occurs in most of the components.

4. Factor 4: *Science*. The positive loadings for this factor emphasise the importance of knowing that treatment is up-to-date and based on the scientific literature (situation 4), understanding the basic science underlying open-heart surgery (situation 8), and a preference for the situations involving radiology (situation 3), the evaluation of scientific evidence (situation 6), and carrying out a research project (situation 7). This factor has been labelled *Science*, although it clearly also contains a component of Research.

Factor scores were extracted for the four factors, and used as the dependent variables in a series of statistical analyses. They are mostly normally distributed, as is seen in Section B of Additional File 1, with a slight skewness for Factor 2 (Helping people), mainly due to a ceiling effect.

### Correlations of background variables with the four factors

The Pearson correlations between the four factors and a range of background measures are provided in Supplementary table 4 of Additional File 1. In view of the large sample size, only results with p < .001 will be mentioned here.

1. Factor 1: *Indispensability*. Participants who rated being indispensable as more important tended to be male, to have lower grades and fewer A*s at GCSE, and to have fewer A*s and fewer points and mean grades in non-science subjects, to have a higher ratio of points in science subjects to non-science subjects, to be more strategic in their study habits, to be less neurotic, more extravert, less agreeable and more conscientious, and to have lower empathy scores on fantasy, perspective-taking, empathic concern and personal distress, as well as lower stress scores.

2. Factor 2: *Helping people*. Participants giving a higher importance to helping people tended to be female, not from ethnic minorities, more agreeable, and higher on the perspective-taking scale of the empathy measure.

3. Factor 3: *Respect*. Participants saying that respect was important to them tended to be younger, to have higher mean grades at GCSE science, to have higher surface learning and lower deep learning scores, to be more neurotic, less open to experience, less agreeable, and less conscientious, to have lower perspective-taking scores, but higher personal distress on the empathy measures, to have lower social desirability scores, and higher stress scores.

4. Factor 4: *Science*. Participants saying that scientific aspects of medicine were important to them tended to be male, from ethnic minorities, to have relatively higher grades at science than non-science GCSEs, to have higher neuroticism and openness scores, and to be less extravert and less agreeable, to have less perspective-taking and more personal distress on the empathy scales, and to have lower social desirability scores.

### Multiple regression

Many background variables are correlated with one another, and therefore it is difficult to interpret the mass of correlations reported in Supplementary table 4 of Additional File 1, and described in the previous section. Multiple regression was therefore used to identify the most important predictors of the four factors. Each factor was in turn used as the dependent variable, and forward entry stepwise regression used to assess the best predictors of the factors. Stepwise regression is known to be overly liberal in its inclusion of variables, and in the present case the sample size is also very large, meaning that quite small correlations can be highly significant. As before, a significance level of p < .001 was chosen, and this was also adjusted by a Bonferroni correction, to take into account the fact that 40 variables were being used. The nominal significance level was therefore set at 0.00001, which also takes adjusts for their being four separate dependent variables. Details of the regression analyses are provided in the Supplementary table 5 in Additional File, with a brief verbal description of the findings being given here.

1. Factor 1: *Indispensability*: Predictors of rating being indispensable as more important were, in order, lower fantasy, personal distress and perspective-taking measures on the empathy scales, higher strategic learning scores, lower grades in non-science GCSEs, and taking more sciences at AS-level. The multiple correlation with the variables in the model was 0.259.

2. Factor 2: *Helping people*. Predictors of a higher importance to helping people were, in order, higher agreeableness scores, being White, and being less extravert. The multiple correlation for these variables was 0.187.

3. Factor 3: *Respect*. Predictors for saying that respect was important were, in order, higher surface learning scores, lower deep learning scores, a lower social desirability score, and higher mean grade at science GCSEs. The multiple correlation was 0.252.

4. Factor 4: *Science*. Predictors for saying that the scientific aspects of medicine were important were, in order, being less agreeable, more open to experience, having greater personal distress, and being male. The multiple correlation was 0.238.

### Social desirability

Social desirability showed significant Pearson correlations with Helping People, where the correlation was positive, and negatively with Respect and Science. However social desirability is in part itself an aspect of personality (which is discussed extensively in the literature on 'lie' scales [43,44]), and that is seen by the fact that in a stepwise multiple regression, high social desirability scores were themselves predicted, in order, by having higher perspective-taking and agreeableness, lower stress, lower neuroticism, and higher conscientiousness. The final significant predictor was lower grades at non-science GCSE grades. Together these variables produced a multiple correlation of .484. In the multiple regressions of the generic motivational factors, social desirability was only significant in predicting Respect, where it was third in importance out of four.

### Ethnic origin

A more detailed breakdown of the generic motivational factors by ethnic origin is provided in Supplementary table 6 of Additional file 1. Differences between the five ethnic grouping are found only for Factor 2 (*Helping people*), and Factor 4 (*Science*). *A posteriori *comparisons show that Chinese/Other and Asian/Asian British applicants each have lower scores than White applicants on *Helping People*, with other groups intermediate. Chinese/Other, Asian/Asian British, and Black/Black British groups each have higher scores on *Science *than the White applicants, with the Mixed group intermediate.

### Path modelling

Although multiple regression can take into account the correlations which exist between predictor variables, it cannot be used to infer the likely causal relations between the measures. Thus, for instance, social class may not apparently have any effect in the multiple correlations because its effect is indirect, via other measures such as learning styles, rather than there being a true independent effect. Such indirect effects are however of importance theoretically and practically. Path analysis (structural equation modelling, causal modelling) provides a solution to the problem by placing variables in a likely causal ordering, which can be justified empirically and theoretically. The technique, which was originally developed in the 1920s by Sewall Wright (see [45]), has been used extensively in the biological, social science and health psychology literature – for overviews see [46-49].

Path models can be complex if they have too many variables. The analysis here therefore is restricted to the four factor measures, the demographic measures of sex, class, medical family and origin, the five personality measures of the big five, the four empathy measures, and the three measures of leaning styles. Most of these variables were significant predictors in the multiple regressions.

### Causal ordering

Path analysis conventionally orders variables from left to right, with those on the left causing those to the right. Demographic variables, such as sex, class, family and ethnic origin are to the extreme left, since they are fixed in the life of the individual. Likewise, personality measures are also relatively fixed, personality by definition and empirically being found to be stable across most of the life-span, and hence the big five measures are to the immediate right of the demographic variables. Learning styles are more variable than personality measures (and can be regarded as half way between states and traits; see Fox *et al *[38]), and hence are to the right of the personality measures. Finally, the four outcome variables are assumed to be determined by the other measures. The measures of empathy are more difficult to place *a priori*, and the situation is complicated by them being fairly highly correlated with the Big Five (see Supplementary table 7 in Additional File 1). We have therefore chosen to express the empathy measures as residuals, after taking the personality variables into account, and then to place them to the right of the personality measures.

Finally, there is the problem of how to place academic achievement. We have chosen to summarise the GCSEs using three measures – the average mean grade at all GCSEs (GCSEmn), the total number of GCSEs taken (GCSEn), and the ratio of points gained from science relative to non-science subjects (GCSEsci). An exploratory factor analysis had confirmed that these variables summarised much of the variance in the various GCSE measures described in Supplementary table 4 of Additional File 1. Placing these three variables on the path diagram is not easy. Clearly they should be to the right of the personality measures, but it is not clear if they cause or are caused by study habits. We have chosen to place the GCSE measures between the personality measures and study habits.

### Significance levels

Just as with multiple regression, there is a risk that many variables and a large sample size will result in paths that are either spurious or too small to be of any real consequence. As before, a significance level of 0.001 was chosen, and then adjusted for the 20 or so variables being entered into the path model, requiring a nominal significance level of 0.0005 for inclusion, equivalent to a t-statistic of 4.05. For reasons of practicality, a value of t≥4 was chosen for inclusion of paths in the model.

### Model fitting

The model is initially fitted as a saturated model in which the associations between all variables at the same level (*i.e*. grouped vertically together in the path diagram) are included in the *Φ *(phi) matrix of LISREL. Causal relations are fitted using the *B *(beta) matrix in LISREL, and paths are included from each variable to all other variables on its right. Once the saturated model is fitted then all *B *paths with t < 2 are removed at one step. At the next step all *B *paths with t < 3 are removed, after which *B *paths are removed one by one, the least significant path being removed at each step until all remaining *B *paths have t ≥ 4. Finally, the modification indices are checked to ensure that none of the excluded sub-diagonal *B *paths would be significant with t ≥ 4 if they were included.

### LISREL model

**Figure 1:**
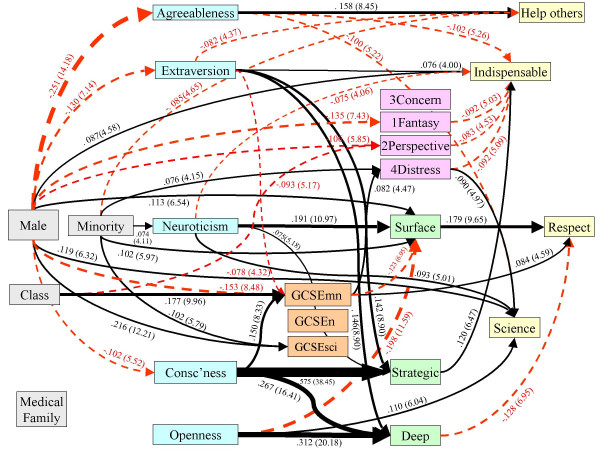
Path diagram showing the relationships between the different variables. Paths are only included for which *t *≥ 4.0. Positive paths are shown as solid black lines, and negative paths as dashed red lines, with path coefficients (path coefficient; *t*) shown in black or red respectively. The thickness of lines is proportional to the size of the path coefficient. Only causal relationships from the *B *(beta) matrix are shown, and can only pass from left to right. Clusters of variables regarded as causally equivalent are shown in the same colour, and in general are vertically above one another, although on some occasions they have been moved slightly to one side or other to make the diagram clearer. Associations between causally equivalent variables are modelled in the *Φ *(phi) matrix, which is saturated within vertical groups and zero elsewhere, and are not shown here (and that in particular explains the apparent absence of a link between being male and being from an ethnic minority). An attempt has been made to avoid lines crossing one another, or lines passing behind variable boxes, but that is not always possible in a diagram of this complexity.

Figure 1 shows the final fitted model which includes all of the variables in the analysis. The fit of the model is very good statistically, with a goodness of fit index of 0.986, an adjusted goodness of fit index of 0.976, and a root-mean-square error of 0.0254. Although the chi-square statistic is not an appropriate test of goodness of fit, since the N is so large, the value of 464.7 with 162 degrees of freedom is very reasonable.

Although the model of figure 1 is at first sight visually complex, like any map it succinctly summarises a large number of relationships. We make no apologies for the complexities, since social phenomena are inherently complex, particularly when indirect effects as well as direct effects are taken into account.

The model contains 45 significant paths, which compares favourably with the 199 paths included in the original saturated model, and many of the paths reflect known and expected relationships between background factors, and therefore help to validate the model, and also indicate indirect effects upon the four main outcome variables. Overall there are seventeen direct effects on the outcome variables (which as it happens is identical to the total number of effects found in the multiple regressions reported in Supplementary table 5 of Additional File 1, although the precise variables included are slightly different). Before considering the model in detail, it is worth briefly mentioning that one variable, Medical Family, has no influences on other variables in the model, and two variables, Number of GCSEs (GCSEn) and Relative GCSE points from Science versus Non-science GCSEs (GCSEsci), have no direct or indirect influences on the four outcome variables (although GCSEsci is influenced by other variables in the model).

**Figure 2:**
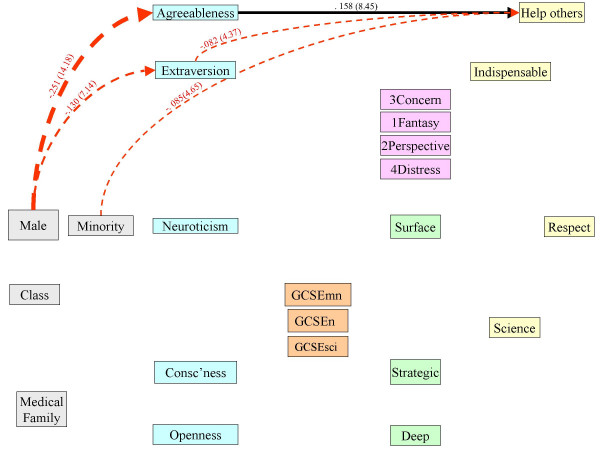
A reduced version of figure 1 in which the only paths shown are those with direct or indirect influences upon generic motivational factor 1 (Indispensability). All variable names have been left in the diagram, and are in the same positions as in figure 1.

**Figure 3:**
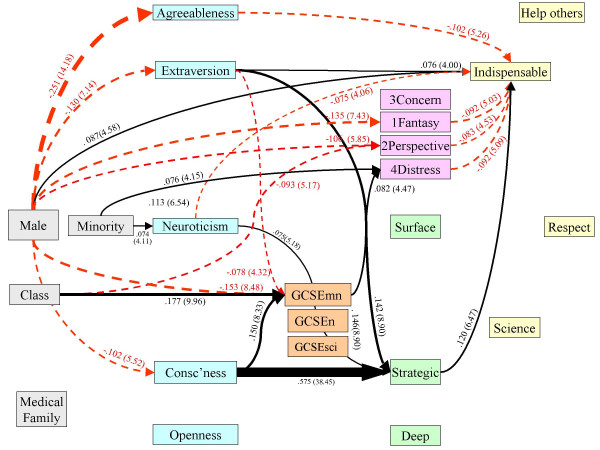
A reduced version of figure 1 in which the only paths shown are those with direct or indirect influences upon generic motivational factor 2 (Helping people). All variable names have been left in the diagram, and are in the same positions as in figure 1.

**Figure 4:**
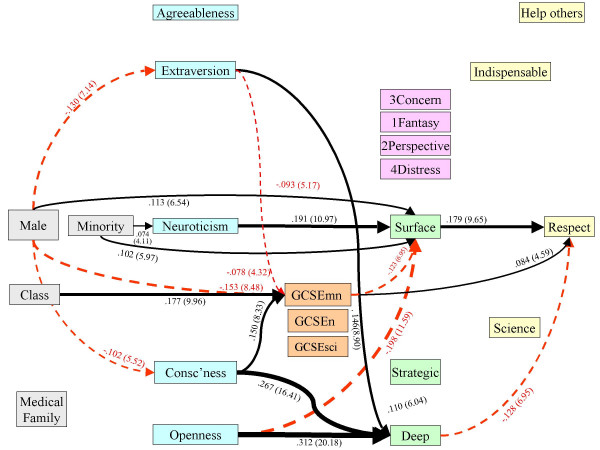
A reduced version of figure 1 in which the only paths shown are those with direct or indirect influences upon generic motivational factor (Respect). All variable names have been left in the diagram, and are in the same positions as in figure 1.

**Figure 5:**
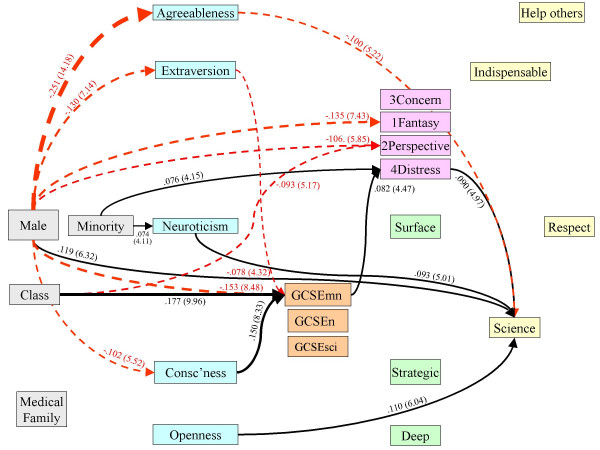
A reduced version of figure 1 in which the only paths shown are those with direct or indirect influences upon generic motivational factor (Science). All variable names have been left in the diagram, and are in the same positions as in figure 1.

The underlying structure of the model can be seen more clearly if figure 1 is simplified, the four outcome variables being considered in turn, and those links removed which do not have either direct or indirect effects upon the outcome variable. These diagrams are seen in figures 2, 3, 4 and 5. Discussion of them will be deferred until the discussion section, since they are best interpreted in terms of known and expected causal relations, and other factors.

## Discussion

This study set out to explore the motivations and interests in a medical career shown by individuals considering applying to medical school, using a novel questionnaire that asks participants to say what particular features of a range of medical scenarios would appeal to them most. Participants found this questionnaire easy and straightforward to complete, with few missing values in the data. Discussion with the participants also suggests that they found it interesting and thought provoking, and appropriate for individuals considering applying to medical school.

Exploratory factor analysis of the Medical Situations Questionnaire found evidence for four clear factors, which were straightforward to interpret, and which we called *Indispensability*, *Helping Others*, *Respect *and *Science*. All had approximately normal distributions, and a range of responses was found which was close to the maximum possible. We believe that these four measures account for much of the variance in the reasons that individuals differ in their motivations for medicine, and in their interests in medicine as a career.

A desirable feature of an assessment of generic motivation is that it is not contaminated with demand characteristics or social desirability. The correlations of the raw scores of the Medical Situations Questionnaire with social desirability are positive for *Helping People*, but are negative for *Respect *and *Science*, and non-significant for *Indispensability*, suggesting that endorsements of the latter three factors are mainly honest self-descriptions. Social desirability is in part itself an aspect of personality – representing a desire to please and an attempt at impression management – and once other personality factors are taken into account in the multiple regression, social desirability correlates only with *Respect*, and then the correlation is negative. The implication is that to a large extent the measures are not contaminated by social desirability or demand characteristics.

If the four motivational and interest factors are valid then it would be expected that they would correlate with a range of background variables in a meaningful and comprehensible way. That there are extensive correlations of the factors is apparent from Supplementary table 5 of Additional File 1, although the multiple regressions suggest that there relatively few predictors for each factor. Exploration of the relationships between the factors and the background measures is seen most clearly in the path analyses, and the four factors will now be considered in detail, using the diagrams in figures 1 to 5.

The reduced path diagram for factor 2, *Helping People *(figure 2) will be considered first, since it is the simplest of the reduced path diagrams. There are three direct paths to the factor which are the same as those found in the multiple regression described in Supplementary table 5 of Additional File 1. Participants who said it was important to help others were more agreeable, less extravert, and were less likely to come from an ethnic minority. The advantages of path analysis over conventional multiple regression are readily seen in the diagram, since it is clear that agreeableness and extraversion are themselves both influenced by sex, the males in this study having lower agreeableness and extraversion scores. However the indirect effect of being male is pulling in two different directions, the lower agreeableness of the males meaning that they should want to help others less, whereas the double negative indirect path via extraversion should mean that males will want to help others more. The path diagram also clarifies many other negative features of the influences on helping others. Thus, whatever the reasons for ethnic minority applicants being less interested in helping others, the effect cannot be explained away in terms of differences in personality, empathy, study habits, academic ability or demography. Neither do any of those variables have any direct influence on wanting to help others, despite *a priori *intuitions that more conscientious, more empathetic, or deeper, more professionally motivated individuals might be more interested in doing so, or that those with particular academic strengths in science might be less interested in doing so. Similar considerations should also be applied when interpreting the path diagrams for the other three factors.

The reduced path diagram for factor 1 (*Indispensability*), Figure 3, is the most complex of the reduced diagrams, with eight background measures having direct effects. Six of these are personality measures, those ranking being indispensable as important being less agreeable, more extravert, less neurotic, and lower on three empathy scores, fantasy, perspective-taking, and distress. In addition they are more strategic in their learning styles, and more likely to be male. Being male has a host of indirect effects, although some of them are 'double negatives', and hence make males less likely to want to be indispensable. The strategic learning style, in which individuals are motivated primarily by a need for success and have study styles designed to achieve examination success, is an important underpinning of indispensability, and is itself driven by conscientiousness. The lower empathy scores of those wanting to be indispensable also suggest a lack of insight, either into the motivations and actions of others, and perhaps also into the motivations of the participants themselves.

The influences on factor 3 (*Respect*), are shown in the reduced path diagram of figure 4, where there are three direct effects. Those motivated by a need for respect tend to have higher surface learning scores, lower deep learning scores, and higher grades at GCSE. The largest effect is from surface learning, for which study habits are driven by a fear of failure and a tendency to resort to rote learning. The second largest effect is the negative path from deep learning, for which study habits are driven by a vocational or professional need or by a desire for understanding, and which therefore result in a *lower *need for respect. Personality has multiple effects, those motivated by a need for respect tending to be more introverted, more neurotic, less conscientious and less open to experience. Ethnic minority participants are show a higher need for respect, both via being more surface learning in approach, and being more neurotic. Being male has multiple influences via many indirect pathways.

Figure 5 shows the reduced path diagram for the influences upon factor 4 (*Science*). There are five direct effects, those motivated or interested more by science and research tending to have higher openness to experience, higher neuroticism, lower agreeableness, more personal distress on the empathy scales, and to be male. Being male also has a range of indirect effects, via a number of intermediate variables. Of particular interest is the combined role of personal distress, neuroticism, neuroticism and disagreeableness, suggesting less a lack of interest in science than a desire to avoid patient contact and the potential emotional traumas of patient contact, and therefore to be a technician or in the backrooms.

The causal influences of variables can also be considered in terms of background variables, rather than outcome variables. Several features are of interest, which are most clearly shown in the overall diagram of figure 1. The overwhelming influence of *Sex *is immediately visible, being male having direct influences on ten of the nineteen variables. Together these factors explain how males are more likely to want to be indispensable, to be scientists, and are less likely to want to help people (see Supplementary table 4 of Additional File 1). Simplistic explanations of sex differences in medicine are belied by the complexity of these relationships. Concerns that the nature of medicine may be changing as the sex ratio alters may in part be justified [16], although it will be towards a medicine more concerned with caring, and less with the need to be indispensable. *Social class *has surprisingly little influence. Its only direct influences are upon perspective-taking, and upon mean GCSE grades, which show a strong linear relation with class (mean grades (standard errors) for social classes I to V: 5.25(.014), 5.13 (.020), 4.95(.037), 5.05(.119) and 4.83(.098)). Academic achievement is well-known to correlate with social class [50-52], and the finding here helps to validate the overall quality both of the academic and social class data in the present study. The encouragement of a wider range of social backgrounds in medical students may have many positive effects, particularly upon equality of opportunity, but is unlikely to result in different motivations for medical practice. *Ethnic origin *has five direct effects, particularly on a lower need to help others and a greater interest in science, higher marks at GCSE science, and higher neuroticism and personal distress. Cross-cultural studies do suggest that there are differences in personality measures, and in particular neuroticism [53]. Some of these differences may partially explain the apparent problems shown in medical schools by ethnic minority medical students, particularly in the clinical part of the course. Finally, and of particular interest in a negative sense, is the measure of *Medical Family*. Despite recurrent suggestions that medical students from medical families are different in their motivation and other aspects of their careers, this study finds no hint that this variable predicts any of our outcome or intermediate measures. The proportion of students from medical families (16.4%) is typical both of entrants and applicants to medical school, and suggests that our participants are representative of medical school applicants, as is also supported by the typical proportions of female (59.2%) and non-white participants (26.1%), which are also broadly compatible with proportions in medical students.

The role of educational variables, in terms of academic achievement, and of the type of subjects studied, is of interest. Despite suggestions that the greater inclusion of medical students whose studies contain less science and more humanities will result in a more caring profession, our study could find no evidence that those studying less science had more interest in helping people. Neither was there any evidence that studying more science led to more interest in the scientific aspects of medicine. Those interested in science in medicine were primarily males and those more open to experience. The only effect of academic qualifications was an indirect effect of higher overall mean grades on a need for respect. It is as if having achieved at a high level produces a desire for recognition because of that fact alone, rather than because of professional achievement as such. Such narcissistic motivations are of interest [54], and would merit further exploration, particularly in qualified doctors. It also has implications for a continuing demand for higher entry standards to medical school (and those standards have changed quite dramatically over the past half-century [55]).

The decision to apply to medical school is a complex one, and applicants and those considering applying have a varied range of interests in a medical career and different motivations. This study has identified what we believe are four major dimensions underlying those interests and motivations – *Helping others*, being *Respected*, being *Indispensable*, and being a *Scientist*. To a fairly large extent these factors can be explained in terms of personality, learning styles, and demographic factors, although no doubt there are manyother events in the lives of our participants, such as individual experience of illness [56], which also make them want to become doctors.

An interesting aspect of the present study is the separation of the interests and motivations, which are generic, from an interest in particular specialities. Although there are some correlations (see Supplementary table 8 in Additional File 1), the overwhelming impression is of the absence of correlation, with the majority of the large correlations (taken as >0.1 or <-.0.1) reflecting the type of a job rather than the specialisation. Thus, *Indispensability *mainly correlates with surgery and acute medical specialities (and not paediatrics, general practice, geriatric medicine, psychiatry or public health), *Science *with pathology and ophthalmology (and not paediatrics or obstetrics), *Respect *only with not being a geriatrician, and *Helping People *with none of the specialities. Other than those broad patterns, the correlations of supplementary table 8 in Additional File 1 are surprisingly weak. To a large extent that probably reflects the fact that all four of the factors can be found in any medical speciality; a surgeon, a psychiatrist, a public health physician and a pathologist can all help people, be respected for what they do, make themselves indispensable, and be a scientist or researcher. The present measures are therefore looking at *what *it is that an applicant most wants to get out of a medical career – what type of experiences they will find rewarding and motivating. *Where *in medicine they find these will depend on a host of other factors, which have been explored elsewhere [57,58] applied to medicine. The key issues are whether one wants to work with people or things, and with ideas or data, but to a first approximation they are probably orthogonal to *Indispensability*, *Helping People*, *Respect *and *Science*.

Most students who eventually enter medical school eventually qualify and have successful careers. However some show a range of problems, both academic and motivational, and a particularly interesting question is whether the sorts of factors we have identified here can also be used either in selecting students, or in counselling them once they are at medical school. However in its present form the Medical Situations Questionnaire is only a research tool, and cannot be used individually, and is not intended for assessing the motivation and interests of individual students, or as the basis for counseling or selection. The alpha reliability coefficients described here are adequate at the group level, but would need to be higher for assessment of individuals. This could be done by increasing the number of scenarios, and broadening their range to increase the generalisability of the questionnaire, as well as by having four choices within each scenario, one for each of the generic motivational factors we have identified. The precise wording of the vignettes could readily be adapted for different contexts, such as studying selection for postgraduate specialities (or even non-medical specialities).

## Competing interests

The author(s) declare that they have no competing interests.

## Authors' contributions

The study was designed jointly by the three authors. ICM was primarily responsible for the design of the questionnaire, and GL and CK were primarily responsible for distribution and collection of the questionnaires and for coding data into the computer. Statistical analysis was primarily the responsibility of ICM. The first draft of the MS was written by ICM, and further drafts, as well as revision of the manuscript was carried out jointly by all three authors.

## Pre-publication history

The pre-publication history for this paper can be accessed here:



## Supplementary Material

Additional File 1 The Medical Situations Questionnaire and additional analyses with supplementary tables and figures. Click here for file
