# Supplementary material for: The attractions of medicine: the generic motivations of medical school applicants in relation to demography, personality and achievement
Source: BMC Med Educ. 2006 Feb 21;6:11. doi: 10.1186/1472-6920-6-11 (PMC1397825; doi:10.1186/1472-6920-6-11)
Supplement: Additional File 1 — The Medical Situations Questionnaire and additional analyses with supplementary tables and figures. [file 1472-6920-6-11-S1.pdf]

The attractions of medicine:

the generic motivations of medical school applicants in relation to demography, personality and achievement

Supplementary File

.

A) *The Medical Situations Questionnaire (four sides).*

B) *Descriptive statistics, factor analysis, and factor scores for the Medical Situations Questionnaire.*

- **Supplementary table 1***:* Percentage of participants rating each item on the Medical Situations Questionnaire as the most appealing or the least appealing.
- **Supplementary figure 1**: Scree-slope for the factor analysis of the Medical Situations Questionnaire.
- **Supplementary table 2**: Factor loadings for each question and situation of the MSQ
- **Supplementary figure 2**: Distributions of factor scores for the four generic motivational factors
- **Supplementary table 3**. Estimated alpha reliability for scores based on factors.

C) Miscellaneous analyses.

- **Supplementary table 4**. Correlations of background variables with the four factors.
- **Supplementary table 5**. Multiple regression of the four factors on the background variables.
- **Supplementary table 6**. Ethnic group differences on the generic motivational factors.
- **Supplementary table 7.** Personality, empathy and stress.
- **Supplementary table 8.** Correlations of generic motivational factors with preferences for specific medical specialities.

Research questionnaire, 2003

This questionnaire is about aspects of medicine as a career, and contains a range of questions about you and your approach to studying and solving problems. For most of the questions there are no right or wrong answers, so please just answer as honestly as possible.

1. Below are nine examples of medical work, followed by three aspects of the doctor's role. Read about each situation, imagine yourself as the doctor, and then indicate how much each aspect appeals to you by putting a 1 next to the *most* appealing, a 2 next to the *next most* appealing, and a 3 next to the *least* appealing.

Situation 1: At 3 a.m. you examine an 18-year old woman in casualty with severe, right-sided abdominal pain, and decide to take her to theatre to remove her inflamed appendix. The next day there are no complications, she returns home and 10 days later you see her to check she is fully recovered.	a) Being the one who makes the decision to operate	
	b) Being on duty in the middle of the night.	
	c) Talking to the patient at follow up and seeing that they have recovered	
Situation 2: In clinic you see a distressed, 21-year old homeless man who is nervous and describes hearing voices. You talk to him about his family, his health and his drug habits. You admit him to hospital compulsorily, arrange appropriate treatment, and then you work with social workers to arrange his discharge into sheltered accommodation. You see him regularly for therapy.	a) Helping to alleviate the patient's social problem	
	b) Being the leader of a team of people helping the patient	
	c) Being respected for your ability to counsel disturbed and difficult patients	
Situation 3: Working in the radiology department you see a man who had been admitted through casualty the night before because of excruciating lower back pain. You perform some special x-rays, examine the films, diagnose kidney stones and write a letter to the GP describing your findings.	a) Knowing that the patient's treatment will depend entirely on your diagnostic skills	
	b) Being in a well-paid, well-respected job with fixed hours	
	c) Knowing that you have helped someone by providing an effective service	
Situation 4: In casualty you see a semi-conscious, 60-year old woman with a chest infection and very high blood sugar levels. You diagnose diabetes, treat her with insulin injections and antibiotics, and monitor her recovery over the next few days. When she feels better you explain to her how diabetes will require treatment for the rest of her life and what is entailed. You then see her every month in outpatients.	a) Treating this patient for a number of years in outpatients or hospital	
	b) Treating an emergency, life-threatening condition in casualty	
	c) Knowing that your treatment is up to date, effective and based on your reading of the latest literature	
Situation 5: In your surgery you see a 50 year old man who has been your patient for the past 10 years. He now reports losing about 5 kg in weight and when you examine him you feel a lump in his abdomen and you suspect cancer. You refer him to hospital and he is found to have an inoperable malignancy. The patient, his wife and son see you frequently over the next 3-4 months, firstly for explanation and then for support. The patient is anxious to avoid going into hospital, and you provide terminal care, pain relief and psychological support, meaning that he dies at home with his family.	a) Playing the most crucial role in diagnosing the patient's condition	
	b) Being respected and trusted by the patient and his family	
	c) Supporting the patient and his family in coping with his death	
Situation 6: You have to evaluate the effectiveness of different methods of screening for breast cancer. You spend several weeks reading scientific literature, visit other health authorities, have discussions with the Department of Health, and go to an international conference. Eventually you chair the meeting at which it is decided to split the budget of £4million pounds between an additional mobile mammography unit and a public awareness campaign.	a) Chairing the meeting at which the crucial decisions are made	
	b) Knowing that the public funds are being used in a way to prevent cancer	
	c) Being responsible for initiating a project, deciding on the best strategies, and steering it to completion	
Situation 7: As part of research project on heart disease, you visit a large number of relatives of a patient with heart disease, collecting blood specimens and interviewing them. You then test the blood samples in the lab, compare the results of this family with others, read about the condition and then write a research paper for *The Lancet*.	a) Being creative, original and working autonomously	
	b) Feeling that you have helped people with heart disease by advancing medical knowledge	
	c) Knowing that you are respected because you publish your results in a top medical journal	
Situation 8: You are an experienced anaesthetist and with the assistance of a junior doctor, nurse, and a theatre technician are responsible for a patient who is undergoing a six-hour open-heart operation to replace a heart-valve. As well as looking after the heart-lung bypass machine, and monitoring the patient's vital functions, you also stay with the patient until they awake on the intensive care unit and their condition is stable.	a) Talking to the patient before the operation, and reassuring them.	
	b) Knowing that you have many technical skills that will always be in demand anywhere	
	c) Understanding the basic science behind the heart-lung machine and the complex monitoring devices	
Situation 9: You are an obstetrician in a busy maternity hospital. In a typical day you may see many patients for routine checks in the ante-natal clinic, you attend four or five deliveries, one of which needed help because progress was too slow and the fetus distressed, you carry out a planned Caesarean section for a case of triplets, and you talk with a mother who has been admitted with a late miscarriage and has lost her baby.	a) Creating a situation in which most women deliver their babies naturally and safely	
	b) Being able to do a Caesarean section	
	c) Knowing how to counsel your patients at an important time of their lives	

Now consider your reaction to the above situations, and imagine how much you would like to be the doctor concerned. In the table below, indicate your preference by putting a *tick* below the <u>three</u> situations you would *most* like to deal with and a *cross* below the <u>three</u> situations with which you would *least* like to be involved.

Situation 1	*Situation 2*	*Situation 3*	*Situation 4*	*Situation 5*	*Situation 6*	*Situation 7*	*Situation 8*	*Situation 9*

2. Below is a list of possible medical careers (it is not meant to be comprehensive). How attractive do you find each of them? Please rate each career from 'Definite intention to go into it' to 'Definite intention *not* to go into it'

	Definite intention to go into this	*Very attractive*	*Not very attractive*	*Definite intention NOT to go into this*
Anaesthetics				
Community child health				
Dermatology (Skin conditions)				
Ear, nose and throat surgery				
Forensic medicine				
General practice				
General surgery				
Genito-urinary medicine				
Haematology (Blood disorders)				
Hospital medicine (e.g. cardiology [heart disease], neurology [disorders of brain & nerve], oncology [cancer treatment])				
Medicine for older people				
Obstetrics and gynaecology				
Ophthalmology (Eye conditions)				
Orthopaedic surgery (Bone & Joint problems)				
Paediatrics (Disorders of children)				
Pathology (e.g. chemical pathology, microbiology)				
Public health				
Psychiatry				
Radiology (Diagnostic imaging)				
Specialist surgery (e.g. neurosurgery, chest surgery)				
Other (please specify)				

3. **Please indicate how far each of the following statements applies to the way in which you approach studying:**

	Rarely true	*Sometimes true*	*True half the time*	*Frequently true*	*Usually true*
i. While I am studying, I often think of real life situations to which the material that I am learning would be useful.					
ii. I chose my present courses largely with a view to the job situation when I graduate rather than their intrinsic interest to me.					
iii. I find that at times studying gives me a feeling of deep personal satisfaction.					
iv. I want top grades in most or all of my courses so that I will be able to select from among the best positions available when I graduate.					
v. I think browsing around is a waste of time, so I only study seriously what's given out in class or in course outlines.					
vi. I try to work consistently throughout the term and review regularly when the exams are close.					
vii. I would see myself basically as an ambitious person and want to get to the top, whatever I do.					
viii. I find that I have to do enough work on a topic so that I form my own point of view before I am satisfied.					
ix. I try to do all of my assignments as soon as possible after they have been set.					
x. I find that studying academic topics can at times be as exciting as a good novel or film.					
xi. I usually become increasingly absorbed in my work the more I do.					
xii. I generally restrict my study to what is specifically set as I think it is unnecessary to do anything extra.					
xiii. I almost resent having to do further years studying after leaving school, but feel that the end results make it all worthwhile.					
xiv. I see getting high marks as a kind of competitive game, and I play it to win.					
xv. I find it best to accept the statements and ideas of my lecturers and question them only under special circumstances.					
xvi. Whether I like it or not, I can see that further education is for me a good way to get a well-paid or secure job.					
xvii. I try to relate new material, as I am reading it, to what I already know on the topic.					
xviii. I keep neat, well organised notes for most subjects.					

4. **Do you agree with the way these statements describe you as a person?**

	Strongly Disagree	*Disagree*	*Neutral*	*Agree*	*Strongly Agree*
I try to be courteous to everyone I meet					
I'm pretty good about pacing myself so as to get things done on time					
When I'm under a great deal of stress, sometimes I feel like I'm going to pieces					
I am intrigued by the patterns I find in art and nature					
I really enjoy talking to people					
I often feel tense and jittery					
I like to be where the action is					
I often feel as if I'm bursting with energy					
I often get angry at the way people treat me					
Some people think of me as cold and calculating					
I have little interest in speculating on the nature of the universe or the human condition					
I generally try to be thoughtful and considerate					
I never seem to be able to get organised					
I often enjoy playing with theories or abstract ideas					
I strive for excellence in everything I do					

5. **Please indicate on the scale provided, how well each of the following statements describes you:**

	Describes me very well	***Describes me fairly well***	***Describes me poorly***	***Describes me very badly***
i. I day dream and fantasise with some regularity about things that might happen to me				
ii. I sometimes find it difficult to see things from another person's point of view				
iii. Sometimes I don't feel very sorry for other people when they are having problems				
iv. In emergency situations I feel apprehensive and ill-at-ease				
v. I try to look at everybody's side of a disagreement before I make a decision				
vi. Becoming extremely involved in a good book or movie is somewhat rare for me				
vii. Other people's misfortunes do not usually disturb me a great deal				
viii. Being in a tense, emotional situation scares me				
ix. I am usually pretty effective in dealing with emergencies				
x. I would describe myself as a pretty soft-hearted person				
xi. I really get involved with the feelings of the characters in a novel				
xii. Before criticising somebody I try to imagine how I would feel if I were in their place				
xiii. I'm always willing to admit it when I make a mistake				
xiv. No matter who I'm talking to, I'm always a good listener				
xv. I sometimes feel resentful when I don't get my way				

6. **How has your health been in general *over the past few weeks*. Have you recently:**

Been able to concentrate on whatever you're doing?	*Better than usual*	*Same as usual*	*Less than usual*	*Much less than usual*
Lost much sleep over worry?	*Not at all*	*No more than usual*	*Rather more than usual*	*Much more than usual*
Felt that you were playing a useful part in things?	*More so than usual*	*Same as usual*	*Less useful than usual*	*Much less useful*
Felt capable of making decisions about things?	*More so than usual*	*Same as usual*	*Less so than usual*	*Much less capable*
Felt constantly under strain?	*Not at all*	*No more than usual*	*Rather more than usual*	*Much more than usual*
Felt that you couldn't overcome your difficulties?	*Not at all*	*No more than usual*	*Rather more than usual*	*Much more than usual*
Been able to enjoy your normal day-to-day activities?	*More so than usual*	*Same as usual*	*Less so than usual*	*Much less than usual*
Been able to face up to your problems?	*More so than usual*	*Same as usual*	*Less able than usual*	*Much less able*
Been feeling unhappy and depressed?	*Not at all*	*No more than usual*	*Rather more than usual*	*Much more than usual*
Been losing confidence in yourself?	*Not at all*	*No more than usual*	*Rather more than usual*	*Much more than usual*
Been thinking of yourself as a worthless person?	*Not at all*	*No more than usual*	*Rather more than usual*	*Much more than usual*
Been feeling reasonably happy, all things considered?	*More so than usual*	*About same as usual*	*Less so than usual*	*Much less than usual*

7. Qualifications: Please put the appropriate grade next to each subject taken at GCSE, AS-level or A-level. Please write 'taking' if the subject is to be taken in the future.

Subject	GCSE grade	Taken/taking AS level	Taken/taking A-level	Subject	GCSE grade	Taken/taking AS level	Taken/taking A level
Biology				German			
Chemistry				History			
Physics				I.T.			
Double science				Latin			
Art				Law			
Dance				Mathematics			
Design & Tech				Music			
Drama				Politics			
Economics				Psychology			
English				Religious			
English				Sociology			
French				Spanish			
Further maths				Other			
Geography				Other			

**8. Have you already taken any degrees? If so, please describe them**	Subject	Level (BA / BSc / MSc/ PhD/ etc)	University	Year of completion	Class

10. Below is the Registrar General's employment classification. Please put ticks next to the categories that best describe your *father*'s occupation and your *mother*'s occupation (if appropriate).

	Mother	*Father*
I Professional - includes doctor, lawyer, architect		
II Managerial - includes shopkeeper, teacher, farmer		
IIINM Non-manual - includes shop assistant, clerical worker		
IIIM Skilled Manual - includes electrician, miner		
IV Partly skilled - includes bus conductors, farm workers		
V Unskilled - includes labourers		

11. Are your parents doctors? *Neither /Mother /Father /Both* **12. Are you** *Male* **or** *Female?* **13. What is your year of birth?** *19*_____

Ethnicity and Religion. It would be helpful to us if you could answer the following questions, which are taken from the **2001 UK Census**.

14. What is your ethnic group? Tick one box to indicate your cultural background.

**White**	**Asian or Asian British**	**Black or Black British**	**Mixed**	**Chinese or Other ethnic group**
British ☐	*Indian* ☐	*Caribbean* ☐	*White & Black Caribbean* ☐	*Chinese* ☐
Irish ☐	*Pakistani* ☐	*African* ☐	*White & Black African* ☐	*Other (please specify)* ☐
Other (please specify) ☐	*Bangladeshi* ☐	*Other (please specify)* ☐	*White and Asian* ☐	
	Other (please specify) ☐		*Other (please specify)* ☐	

15. What is your religion? Please tick one box.

None ☐ *Christian* ☐ *Buddhist* ☐ *Hindu* ☐ *Jewish* ☐ *Muslim* ☐ *Sikh* ☐ *Other (please specify)* ☐

Thank you for completing this detailed questionnaire and helping with the survey

As described in the covering letter, this questionnaire is entirely for the purposes of research. It is entirely confidential and will be stored safely and electronic versions will be stored in an anonymised form. None of the information provided will be used for the purposes of selection, and nor will it be provided to those examining or teaching students. Results from the survey will only be published in aggregate or other form in which individuals cannot be identified. Both for your own reassurance and as a normal part of research ethics, **we would be grateful if you could consent to the information being used for this purpose by initialing here:**

Initials ___________ *Date* ____/_____/_______

In order that we can link these data with other information, and provide feedback on the survey, it is important that you provide us with contact details in BLOCK CAPITAL. As with the rest of this questionnaire, your provision of this information and your participation are optional.

Name:

Home Address:

E-mail address:

b) *Descriptive statistics, factor analysis, and factor scores for the Medical Situations Questionnaire.*

Supplementary table 1: Percentage of participants rating each item on the Medical Situations Questionnaire as the most appealing or the least appealing.

		% Most appealing	% Least appealing
Scenario 1 (Surgery)	Making the decision to operate	39.7	15.1
	Being on duty at night	4.7	73.8
	Talking to the patient at follow-up	55.6	11
Scenario 2 (Psychiatry)	Alleviating social problems	59	16.3
	Being the leader of the a team	20.2	40.8
	Being respected for counselling ability	20.9	42.9
Scenario 3 (Radiology)	Diagnostic skills	15.2	27.6
	Well-paid, well-respected job	5.9	68.4
	Helping by providing effective service	79	3.9
Scenario 4 (Hospital medicine)	Treating patient over several years	17.7	47.6
	Treating an emergency condition	51.9	20
	Knowing treatment is effective	30.5	32.4
Scenario 5 (General practice)	Playing crucial role in diagnosis	15.3	59.5
	Being respected by patient and family	42.6	18.1
	Supporting terminally-ill patient	42.3	22.3
Scenario 6 (Public health)	Chairing the crucial meeting	6.2	73.4
	Using public funds effectively	54.9	15.3
	Initiating and steering the research project	39.1	11.3
Scenario 7 (Research)	Creative, autonomous work	13.4	25.3
	Helping by advancing medical knowledge	82.9	3.2
	Respect for publishing in medical journals	3.8	71.5
Scenario 8 (Anaesthetics)	Reassuring patient pre-operatively	55.8	13.4
	Complex, much-needed technical skills	18.6	56
	Applying basic medical science	25.7	30.6
Scenario 9 (Obstetrics & Gynaecology)	Providing a safe, caring environment	48.1	16.5
	Knowing how to do a Caesarean section	15.9	58.9
	Counselling patients	36.1	24.6
Scenario preferences	Working in surgery	53.1	12.7
	Working in psychiatry	30.4	28.5
	Working in radiology	18.5	28.6
	Working in hospital medicine	36.3	13.7
	Working in general practice	33.8	31
	Working in public health	16.5	49.8
	Working in research	18.7	47.2
	Working in anaesthetics	41.2	21
	Working in obstetrics and gynaecology	37.8	30.9

For the scenarios the columns show the percentage of respondents saying a particular aspect of the work was most or least appealing. For the preferences for the scenarios, the columns show the percentages of respondents including that scenario as one of their three most preferred or three least preferred scenarios, so percentages do not sum to 100%. Because of occasional missing values, N for the preferences within scenarios varies from 2830 to 2849.

Supplementary figure 1: Scree-slope for the factor analysis of the Medical Situations

Questionnaire.

Technical comment: It should be noted that the scree-plot is somewhat unusual in that the last eigenvalues are effectively zero. This reflects the fact that for the preferences within each of the nine scenarios there are strong contingencies because of the ranking of the three choices. Although each choice is allocated a score from 3 (most preferred) to 1 (least preferred), these scores are necessarily linearly related in the sense that once the first two judgements are made then there is no choice over the third. As a result the theoretical values for the last nine eigenvalues are all zero. There is also a minor degree of constraint between the preferences for the scenarios, but it has relatively little impact on the overall eigenvalues.

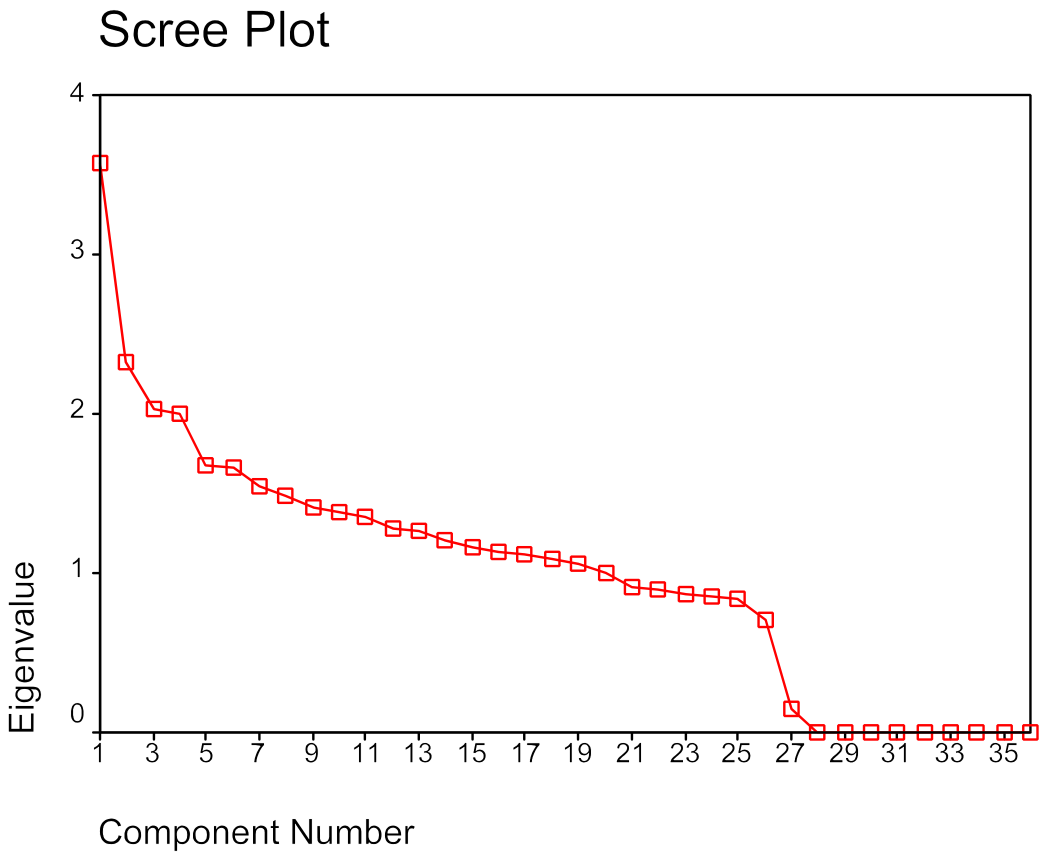

Supplementary table 2: Factor loadings for each question and situation of the MSQ

		Factor 1 Indispensability	Factor 2 Helping people	Factor 3 Respect	Factor 4 Science
Scenario 1 (Surgery)	Making the decision to operate	**0.537**	-0.175	-0.069	-0.214
	Being on duty at night	-0.014	-0.186	0.044	0.091
	Talking to the patient at follow-up	-0.539	0.33	0.034	0.147
Scenario 2 (Psychiatry)	Alleviating social problems	-0.236	**0.474**	-0.281	0.003
	Being the leader of the a team	0.311	-0.235	-0.162	0.126
	Being respected for counselling ability	-0.073	-0.236	**0.435**	-0.127
Scenario 3 (Radiology)	Diagnostic skills	0.235	-0.317	**-0.45**	-0.222
	Well-paid, well-respected job	-0.16	-0.11	**0.6**	0.3
	Helping by providing effective service	-0.108	**0.523**	-0.132	-0.068
Scenario 4 (Hospital medicine)	Treating patient over several years	**-0.429**	-0.061	0.079	-0.179
	Treating an emergency condition	**0.422**	-0.16	-0.102	**-0.359**
	Knowing treatment is effective	-0.012	0.213	0.024	**0.526**
Scenario 5 (General practice)	Playing crucial role in diagnosis	**0.46**	-0.246	-0.117	0.216
	Being respected by patient and family	-0.094	0.037	**0.472**	-0.001
	Supporting terminally-ill patient	**-0.351**	0.199	-0.335	-0.203
Scenario 6 (Public health)	Chairing the crucial meeting	0.148	**-0.451**	0.155	0.011
	Using public funds effectively	-0.068	**0.629**	0.064	0.005
	Initiating and steering the research project	-0.053	-0.309	-0.211	-0.014
Scenario 7 (Research)	Creative, autonomous work	-0.261	-0.344	**-0.424**	0.337
	Helping by advancing medical knowledge	0.168	**0.543**	-0.138	-0.283
	Respect for publishing in medical journals	0.149	-0.092	**0.596**	-0.128
Scenario 8 (Anaesthetics)	Reassuring patient pre-operatively	**-0.443**	0.103	-0.238	**-0.416**
	Complex, much-needed technical skills	0.212	-0.161	**0.511**	0.031
	Applying basic medical science	0.204	0.069	-0.304	0.367
Scenario 9 (Obstetrics & Gynaecology)	Providing a safe, caring environment	0.115	**0.403**	-0.003	0.13
	Knowing how to do a Caesarean section	**0.425**	-0.279	0.169	0.118
	Counselling patients	**-0.525**	-0.112	-0.162	-0.24
Scenario preferences	Working in surgery	0.325	0.15	-0.021	-0.129
	Working in psychiatry	**-0.388**	-0.055	-0.038	-0.16
	Working in radiology	0.145	0.021	0.104	0.258
	Working in hospital medicine	-0.001	0.039	0.006	-0.201
	Working in general practice	-0.17	-0.099	-0.069	-0.324
	Working in public health	-0.047	-0.096	-0.017	**0.44**
	Working in research	-0.049	-0.146	-0.029	**0.499**
	Working in anaesthetics	0.292	0.073	0.018	-0.048
	Working in obstetrics and gynaecology	-0.036	0.105	0.077	-0.294

Negative loadings are shown in red, and loadings in small type have an absolute value of less than .2. Absolute loadings higher than 0.35 are in bold, and absolute loadings higher than 0.5 are underlined.

Supplementary figure 2: Distributions of factor scores for the four generic motivational factors, calculated directly from the factor analysis (left), and from raw scores (right).

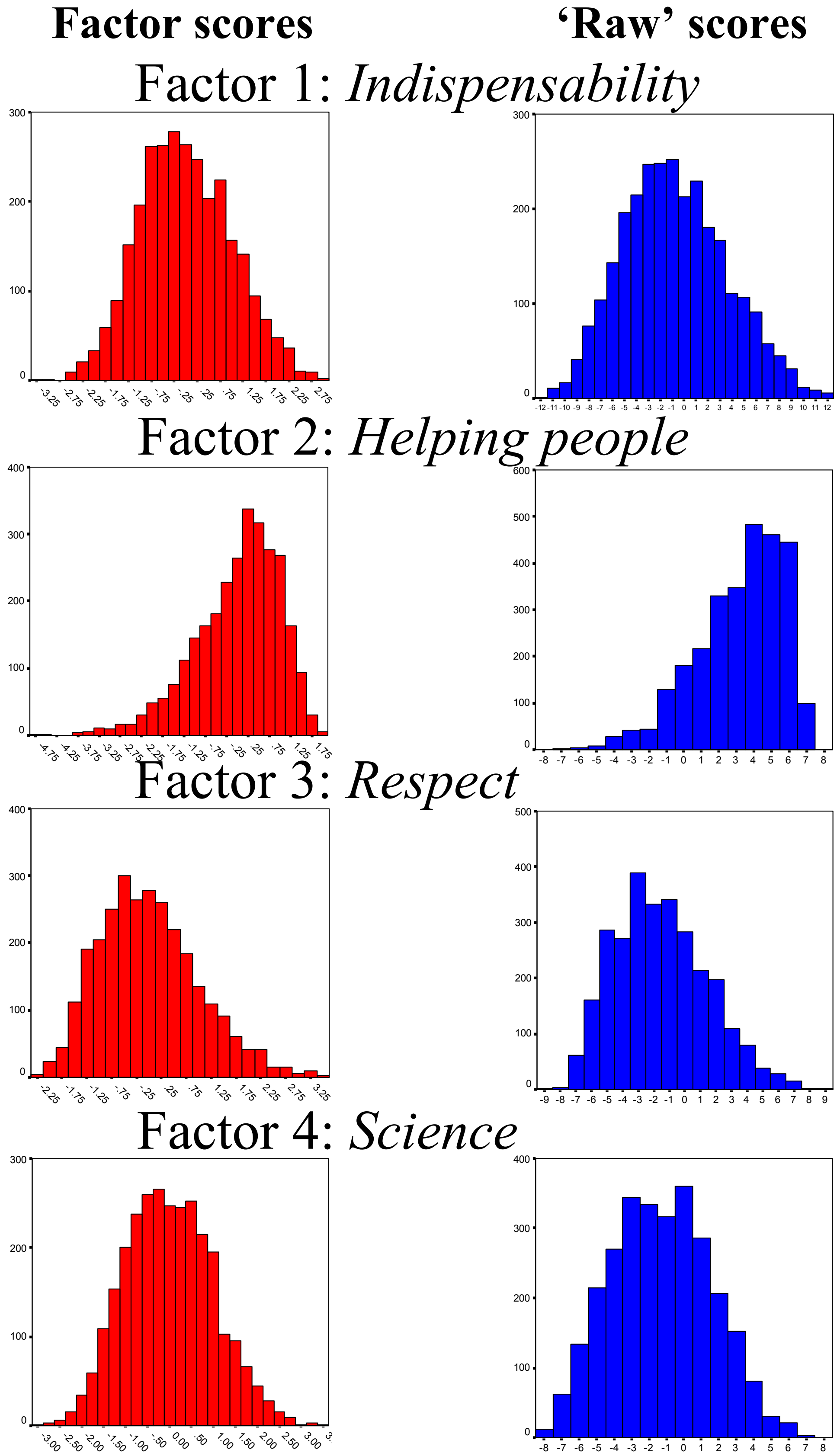

Supplementary table 3. Reliability of scores based on factors.

The reliability of factor scores cannot be calculated directly. However a standard method is to base a scale on the most heavily loading raw items, and to use that to estimate coefficient alpha. Items were reverse scored when loadings in the factor analysis were negative. Items labelled as 1a, 3b, etc refer to the preferences within the scenarios, whereas items labelled as 1, 3, etc refer to preferences for the scenarios themselves. Items were chosen for the four scales so that, as far as possible, one item was used for each scale. In four cases (5c, 8a, 7a, 8c) the items were allowed to load on several scales, since they had high and near equal loadings on the scales, and in one case (8c) the item was reverse scored on one scale and normally scaled on the other.

	Items included in scale (* = reversed scoring)	N(items)	Cronbach's alpha
Score 1: Indispensability	1a, 1c*, 2b, 4a*, 4b, 5a, 5c*, 8a*, 9b, 9c*, 1, 2*, 8	13	0.671
Score 2: Helping people	2a, 3c, 6a*, 6b, 6c*, 7a*, 7b, 9a	8	0.537
Score 3: Respect	2c, 3a*, 3b, 5b, 5c*, 7a*, 7c, 8b, 8c*	9	0.58
Score 4: Science	4c, 8a*, 8c, 3, 5*, 6, 7, 9*	8	0.507

If one wishes to generate scores for the various scales, then score each first ranked choice within scenarios as +1, each second ranked choice as 0 and each third ranked choice as -1, and for the choice between scenarios scores +1 for each scenario indicated as most liked, -1 for each scenario indicated as least liked, and zero for other scenarios.

Supplementary table 4. Pearson correlations of generic motivational factors with background measures. * p<.05; ** p<.01; *** p<.001. Sample sizes are not provided, to avoid undue complexity, but vary between 2601 and 2867 for all variables, except the four AS-level variables for which sample size is 2262.

	Factor 1 *Indisp-ensability*	Factor 2 *Helping people*	Factor 3 *Respect*	Factor 4 *Science*
Male (1=male, 0=female)	.116 ***	-.069 ***	-.025	.137 ***
Age	-.008	-.022	-.078 ***	-.002
Ethnic origin (1=minority 0 =White)	-.040 *	-.097 ***	-.016	.079 ***
Mother a doctor (1=Yes 0= No)	-.028	-.043 *	.016	.016
Father a doctor (1=Yes 0= No)	-.011	.004	.020	.026
Medical parent(s) (1=Yes 0= No)	-.014	-.014	.005	.030
Social class (I=5, II=4, III=3, IV=2, V=1)	-.011	-.048 *	.031	.007
Number of GCSEs taken	.003	.055 **	.033	.019
Points at GCSE	-.049 **	.028	.057 **	-.010
A* grades at GCSE	-.070 ***	-.022	.054 **	-.026
Mean grade at GCSE (6=A*, 5=A ... 1=E)	-.079 ***	-.017	.053 **	-.037
Number of science GCSEs taken	.056 **	-.003	-.032	-.003
Points at GCSE sciences	.037	.000	.028	-.002
A* grades at GCSE sciences	-.015	-.024	.054 **	-.011
Mean grade at GCSE science	-.038 *	-.006	.072 ***	-.003
Number of non-science GCSEs taken	-.029	.036	.003	-.013
Points at GCSE non-sciences	-.082 ***	.018	.030	-.017
A* grades at GCSE non-sciences	-.094 ***	-.014	.035	-.032
Mean grade at GCSE science	-.097 ***	-.018	.035	-.049 **
Ratio science to non-science GCSEs taken	.055 **	-.049 **	-.019	.005
Ratio science to non-science GCSE points	.083 ***	-.027	-.014	.034
Ratio science to non-science mean grades	.060 **	.004	.023	.065 ***
Number of AS levels	.000	-.051 *	-.011	.036
Number of AS level sciences	.111 ***	-.058 **	.008	.101 ***
Number of AS level non-sciences	-.112 ***	.030	-.014	-.081 ***
Ratio science to non-science AS levels	.117 ***	-.040	.012	.095 ***
Big Five - Neuroticism	-.072 ***	-.036	.096 ***	.102 ***

	Factor 1 *Indisp-ensability*	Factor 2 *Helping people*	Factor 3 *Respect*	Factor 4 *Science*
Big Five - Extraversion	.082 ***	-.049 **	-.064 ***	-.095 ***
Big Five - Openness to experience	-.050 **	-.007	-.124 ***	.101 ***
Big Five - Agreeableness	-.072 ***	.149 ***	-.088 ***	-.136 ***
Big Five - Conscientiousness	.074 ***	-.009	-.101 ***	-.047 *
Stress (GHQ-12, 0-1-2-3 scoring)	-.110 ***	-.007	.096 ***	.044 *
Empathy scale – Fantasy	-.127 ***	.053 **	-.003	.032
Empathy scale – Perspective-taking	-.084 ***	.073 ***	-.126 ***	-.069 ***
Empathy scale – Empathic concern	-.092 ***	.007	-.015	-.047 *
Empathy scale – Personal distress	-.123 ***	.033	.143 ***	.129 ***
Surface Learning	.028	-.055 **	.184 ***	.034
Deep Learning	.037	-.055 **	-.151 ***	.002
Strategic Learning	.108 ***	-.031	-.037	.014
Social Desirability scale	-.030	.062 ***	-.142 ***	-.094 ***

Supplementary table 5. Regression of generic motivational factors on background variables. Degrees of freedom are not given, but in all cases are extremely large, so that *t* statistics are asymptotically equivalent to *z* statistics. *t* statistics, beta coefficients and significance levels are given for the final step of the regression, and hence effects for each take into account for all other variables in the model. Significance levels have not been subject to the Bonferroni correction. **Note:** The empathy measures in this analysis are raw scores, as in table 1; however in the LISREL model they are residuals after removing effects of the Big Five.

Dependent variable	Stepwise predictors in order		Beta	t (sig)
Factor 1: *Indispens-ability*	1	Empathy - Fantasy	-.110	t= -6.048 (p= 1.4x10^{-9})
	2	Empathy - Personal distress	-.128	t= -6.858 (p= 6.9x10^{-12})
	3	Empathy - Perspective-taking	-.115	t= -6.171 (p= 6.8x10^{-10})
	4	Strategic learning	.107	t= 5.785 (p= 7.3x10^{-9})
	5	Mean GCSE grade for non-science subjects	-.092	t= -5.037 (p= 4.7x10^{-7})
	6	Number of sciences at AS-level	.084	t= 4.631 (p= 3.6x10^{-6})
Factor 2: *Helping People*	1	Agreeableness	.155	t= 8.280 (p< 10^{-12})
	2	Non-white ethnicity	-.086	t= -4.690 (p= 2.7x10^{-6})
	3	Extraversion	-.082	t= -4.394 (p= 1.1x10^{-5})
Factor 3: *Respect*	1	Surface learning	.160	t= 8.627 (p<10^{-12})
	2	Deep learning	-.104	t= -5.260 (p=1.9 x10^{-8})
	3	Social desirability	-.098	t= -5.287 (p= 1.2x10^{-7})
	4	Mean GCSE grade for science subjects	.078	t= 4.264 (p= 2.0x10^{-5})
Factor 4: *Science*	1	Agreeableness	-.104	t= -5.488 (p= 4.1x10^{-8})
	2	Openness to experience	.126	t= 6.857 (p= 7.0x10^{-12})
	3	Personal distress	.129	t= 6.953 (p= 3.6x10^{-12})
	4	Male sex	.104	t= 5.602 (p= 2.1x10^{-8})

Supplementary table 6. Ethnic group differences on the generic motivational factors. The overall ANOVA result is shown at the top of each section. Groups are ordered from the lowest to the highest mean, and superscripts indicate groups which are not statistically different using the Ryan-Einot-Gabriel-Welsch range statistics for *a posteriori* comparisons. No groups are significantly different using that criterion for factor 1 and factor 3.

Factor 1: *Indispensability* (F(4,2822) = 3.03, p = .017)

	Black or Black British	Asian or Asian British	Mixed	White	Chinese or Other
Mean	-0.177	-0.119	-0.063	0.021	0.132
SD	1.04	0.891	1	1.022	0.947
N	58	455	80	2090	144
Factor 2: *Helping people* (F(4,2822)=7.698, p <.001)					
	Chinese or Other	Asian or Asian British	Black or Black British	Mixed	White
Mean	-.252[a]	-.170[a]	-.143[a,b]	.029[a,b]	.057[b]
SD	1.06	1.108	0.799	1.111	0.057
N	144	455	58	80	2090
Factor 3: *Respect* (F(4,2822)=0.366, p = 0.833)					
	Chinese or Other	Asian or Asian British	Black or Black British	White	Mixed
Mean	-0.081	-0.026	-0.019	0.007	0.038
SD	0.983	1.077	0.887	0.987	1.04
N	144	455	58	2090	80
Factor 4: Science (F(4,2822)= 5.412, p <.001)					
	White	Mixed	Chinese or Other	Asian or Asian British	Black or Black British
Mean	-.050[a]	-.003[a,b]	.041[a,b]	.167[b]	.241[b]
SD	1.017	1.001	0.041	0.94	1.002
N	2090	80	144	455	58

Supplementary table 7. Personality, empathy and stress.

The table below shows that the four empathy measures are strongly correlated with the fiver personality measures, as might be expected. Including such relationships in the path diagrams of the main paper would complicate the diagram and hence the empathy measures have been included as residuals after partialling out the effects of all five personality components, using multiple regression. The Pearson correlations between all of the measures are shown below.

Although stress levels, using the 0-1-2-3 scoring of the GHQ-12 were included in the study, they were not included in the path diagrams because of a need to reduce complexity. They are however of some theoretical interest, not least because of the large sample size, the fact that the relationships are present in individuals who are not at medical school but still at school, and because they closely replicate the findings one of us has reported elsewhere, that high stress levels correlate with higher neuroticism levels, lower extraversion, lower agreeableness and lower conscientiousness (and also, as found before, there is no correlation with openness to experience). Stepwise multiple regression with a 0.001 inclusion level found that neuroticism, conscientiousness and extraversion were significant predictors, in that order.

Sample sizes vary from 2637 to 2748. * p<.05; ** p<.01; *** p<.001.

	Neuroticism	Extraversion	Openness	Agreeableness	Conscien-tiousness
Empathy: Fantasy	.116 ***	.085 ***	.275 ***	.089 ***	-.081 ***
Empathy: Perspective-taking	-.205 ***	.117 ***	.201 ***	.343 ***	.193 ***
Empathy: Empathic concern	.166 ***	-0.006	0.013	.105 ***	-.048 *
Empathy: Personal distress	.339 ***	-.336 ***	-.156 ***	-.145 ***	-.226 ***
Stress (GHQ12, 0-1-2-3 scoring)	.416 ***	-.162 ***	0.021	-.160 ***	-.268 ***

Supplementary table 8. Pearson correlations of generic motivational factors with interest in specific medical specialities. * p<.05; ** p<.01; *** p<.001. Sample sizes are not provided, to avoid undue complexity, but vary between 2750 and 2810.

	Factor 1 Indisp-ensability	Factor 2 Helping people	Factor 3 Respect	Factor 4 Science
Anaesthetics	.165 ***	.074 ***	0.034	-0.01
Community child health	-.193 ***	.072 ***	-0.02	-.086 ***
Dermatology (Skin conditions)	-0.01	-0.029	0.004	.063 ***
Ear, nose and throat surgery	.136 ***	-0.016	-0.01	0.031
Forensic medicine	0.04	-.054 **	-0.01	.058 **
General practice	-.120 ***	.038 *	0.019	.055 **
General surgery	.222 ***	-0.014	-0.03	-0.023
Genito-urinary medicine	0.002	0.009	-0.045	0.035
Haematology (Blood disorders)	-0.018	-0.008	-0.053	.088 ***
Hospital medicine (e.g. cardiology [heart disease], neurology [disorders of brain & nerve], oncology [cancer treatment])	.106 ***	0.01	-.086 ***	-.067 ***
Medicine for older people	-.149 ***	.062 ***	-.117 ***	-.063 ***
Obstetrics and gynaecology	-.044 *	.039 *	-0.01	-.109 ***
Ophthalmology (Eye conditions)	0.02	-0.017	0.019	.136 ***
Orthopaedic surgery (Bone & Joint problems)	.094 ***	0.028	0.01	0.024
Paediatrics (Disorders of children)	-.115 ***	.085 ***	-0.023	-.203 ***
Pathology (e.g. chemical pathology, microbiology)	.049 **	-.052 **	-.044 *	.115 ***
Public health	-.122 ***	0.036	-.062 ***	0.012
Psychiatry	-.219 ***	-.074 ***	0	0
Radiology (Diagnostic imaging)	.053 **	0.014	.077 ***	.146 ***
Specialist surgery (e.g. neurosurgery, chest surgery)	.265 ***	-.060 ***	-.052 **	-0.021
